# Advancements in SELEX Technology for Aptamers and Emerging Applications in Therapeutics and Drug Delivery

**DOI:** 10.3390/biom15060818

**Published:** 2025-06-05

**Authors:** Liangjie Feng, Yu Sun, Wenshen Jia, Yang Yu, Chang Liu, Jing Yang, Yunxia Luan, Jin Chen, Fengchao Wang

**Affiliations:** 1 College of Sciences, Shanghai Institute of Technology, 100 Haiquan Road, Shanghai 201418, China; 2 Institute of Quality Standard and Testing Technology of BAAFS, Beijing 100097, Chinaluanyx@iqstt.cn (Y.L.)

**Keywords:** aptamer, SELEX, targeted drug delivery, therapeutics, nanotechnology, conjugates

## Abstract

Nucleic acid aptamers, selected through the Systematic Evolution of Ligands by Exponential Enrichment (SELEX), are short nucleic acid sequences that exhibit high affinity and specificity towards diverse targets. Over the past three decades, substantial advancements have been made in both the technology and applications of nucleic acid aptamers. This review provides an in-depth analysis of the historical development and defining characteristics of aptamers, highlighting recent technological innovations in SELEX, including Capillary Electrophoresis SELEX, Microfluidic SELEX, Cell-SELEX, and others. We explore the applications of aptamers in therapeutic and targeted drug delivery, emphasizing their advantages over traditional antibodies such as cost-effectiveness, ease of synthesis, and lower immunogenicity. Key challenges such as stability, specificity, and efficient delivery are discussed, with proposed strategies for improvement including advanced chemical modifications and integration with nanotechnology. By integrating advanced technologies, aptamers hold significant promise for enhancing precision medicine and personalized therapeutic interventions, offering new avenues for the treatment of complex diseases.

## 1. Introduction

Nucleic acid aptamers, generated through the Systematic Evolution of Ligands by Exponential Enrichment (SELEX), are functional nucleic acids known for their specificity and high affinity. Typically, aptamers are short single-stranded DNA or RNA sequences [1]. Aptamers are often compared to “chemical equivalents of antibodies” due to their ability to specifically recognize targets with high affinity. Unlike protein antibodies (Table 1), aptamers offer several advantages [2]: they can be easily synthesized chemically in vitro, are relatively cost-effective, and typically exist in stable powder form. Additionally, aptamers can be stored at room temperature or 4 °C unlike antibodies that require extremely low temperatures, enhancing their convenience for acquisition and use. Thousands of aptamers have been successfully designed to recognize a wide array of targets, including metal ions [3], organic molecules [4], proteins [5], and whole cells [6]. Furthermore, nucleic acid aptamers can be chemically modified with luminescent groups, dyes, and enzymes to build aptamer sensors [7], finding applications in environmental monitoring, food safety, disease diagnostics, and drug delivery.

The SELEX method was initially introduced by Tuerk and Gold in 1990 [8]. Since then, SELEX technology has been continuously evolving, combining a series of advanced technological means, such as CE-SELEX [9], Microfluid SELEX [10], Cell-SELEX [11], Capture SELEX [12], In-Silicon SELEX [13], Deep Sequencing SELEX [14], etc. These technologies make SELEX more efficient, precise, and customizable. So far, the number of aptamers that exhibit strong binding ability and specificity is still relatively small. Improving existing SELEX programs and developing more aptamers with high binding ability and specificity remain the main obstacles in basic and applied research related to aptamers. Notably, aptamers typically assume specific structural conformations due to their propensity for forming complementary base pairs. There are a variety of secondary structures for aptamers, such as stem-loop, hairpin, G-quadruplex, pseudoknot, and kissing-loop structures [15]. However, the integration and accumulation of these technologies will promote the further development of SELEX technology, enabling it to demonstrate greater potential in biomedical research and applications.

**Figure 1:**
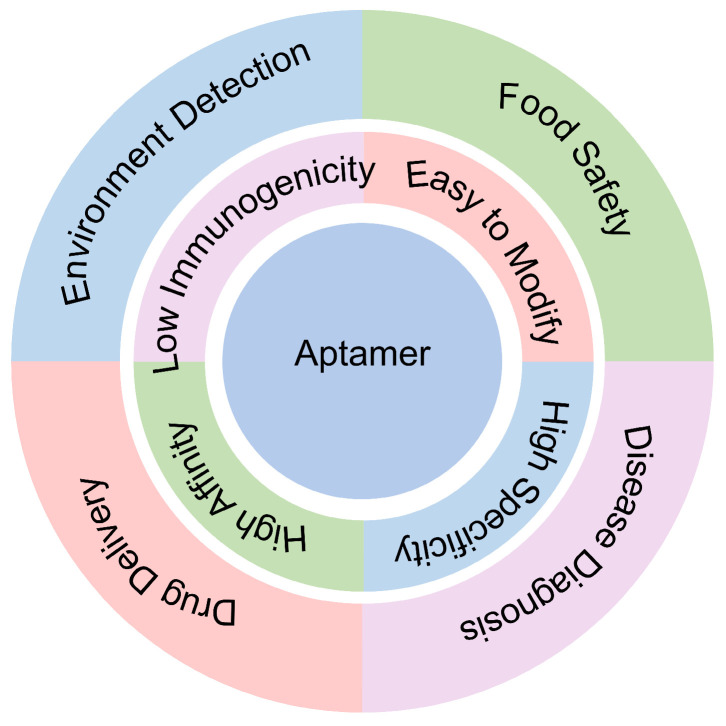
The advantages and applications of nucleic acid aptamers.

Over the past three decades, nucleic acid aptamers have demonstrated significant potential in the field of therapeutics [16,17]. Aptamers meticulously selected through SELEX technology exhibit high specificity in binding to biomarkers such as proteins and cell surface receptors, offering a novel avenue for early disease detection. This high specificity also positions nucleic acid aptamers as ideal components of biosensors for detecting pathological molecules, thus enabling more sensitive diagnostic methods. Additionally, nucleic acid aptamers play a crucial role in drug delivery [18]. Their highly specific binding properties with target molecules provide a reliable platform for precise drug delivery. By conjugating drugs with nucleic acid aptamers, targeted drug delivery can be achieved, minimizing adverse effects on normal tissues and enhancing therapeutic efficacy (Figure 1). This targeted delivery approach also helps surmount challenges encountered by traditional drug delivery methods, including drug instability and side effects. This review provides an overview of the latest advancements and challenges in SELEX technology and explores the applications of nucleic acid aptamers in therapeutics and drug delivery with particular emphasis on the various challenges encountered in practical clinical settings.

## 2. Enhancing SELEX Methodology: Innovations and Refinements

### 2.1. SELEX Technology

**Figure 2:**
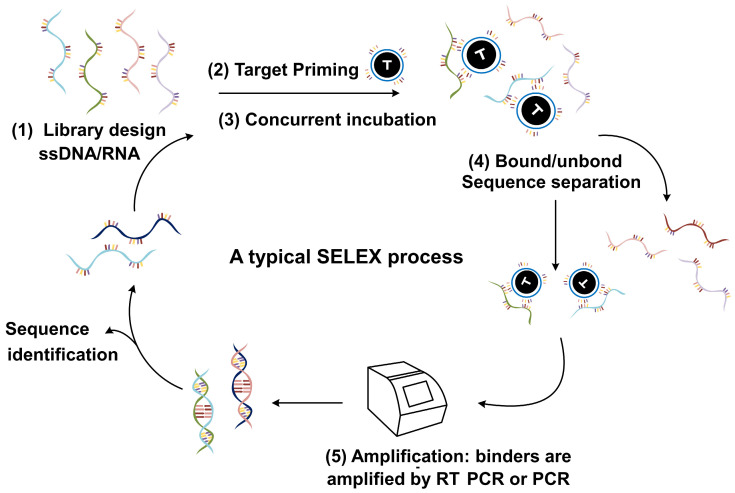
A typical SELEX process.

SELEX technology [19], also known as the Systematic Evolution of Ligands by Exponential Enrichment, is a powerful and flexible method for in vitro screening of high-affinity nucleic acid aptamers (Figure 2). The basic principle involves screening oligonucleotide sequences that can bind highly specifically to target molecules. This process is carried out through multiple rounds of in vitro evolutionary cycles from the initial oligonucleotide library. The key steps of this process include the initial oligonucleotide library, binding selection, elution, amplification with the target molecule, and multiple cycles. The initial oligonucleotide library contains a large number of random sequences, which selectively retain the nucleotide sequences bound to the target molecule through reactions. The subsequent elution and PCR amplification steps help increase binding affinity and create more favorable conditions for the next round of selection. This multi-cycle process gradually enriches the specificity and affinity of nucleic acid aptamers, ultimately obtaining nucleic acid aptamers with highly specific binding ability.

SELEX is a powerful and effective selection approach. Although the classic SELEX has been proven to have vital potential in adaptive selection, significant endeavors have been made to simplify the method and improve the SELEX selection efficiency [20]. These efforts include improving SELEX’s overall performance, such as via efficient recycling and sorting, precise amplification, and comprehensive data sequencing processing analysis. In addition, specialized analytical techniques, such as Capillary Electrophoresis SELEX, Cell-SELEX, and Microfluid and Microarray SELEX, have been successfully applied in classical SELEX programs to accelerate the identification of high-affinity aptamers. For example, the traditional SELEX process is considered to be conducted in a “black box” before the final round, meaning that the details of the process are not visible until a specific aptamer is determined. However, the introduction of next-generation sequencing (NGS) has changed this situation, making the selection process observable in every round, thereby fundamentally transforming the process of aptamer selection.

### 2.2. Advances in SELEX Techniques

#### 2.2.1. Capillary Electrophoresis SELEX

**Figure 3:**
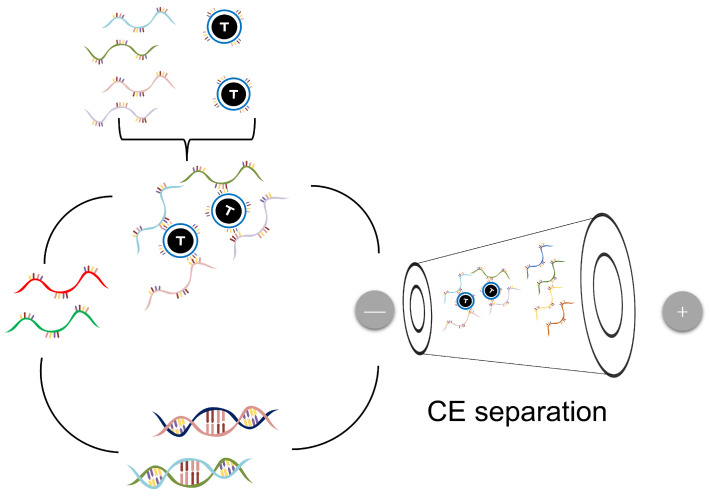
The CE-SELEX process.

In 2004, capillary electrophoresis (CE) was used for ligand screening using a high-voltage electric field as the propelling force based on differences in component mobility [21] (Figure 3). Compared to the filters and affinity chromatography methods used by early biologists [8,22], CE provides an effective method for separating and collecting complex, high-resolution complexes and unbound single-stranded DNA. CE is not only a separation tool but also an analytical technique that can be used to determine affinity constants and help monitor both the enrichment of single-stranded DNA in each round of selection as well as the purity or quality of target substances and single-stranded DNA libraries. As a microanalysis technique, CE has a lower cost and allows for more experiments compared to other methods, using the same amount of reagents and samples. In addition, CE also has multiple separation modes that can be flexibly applied to achieve different functions. Throughout the entire SELEX process, CE plays multiple roles and is widely acknowledged for its exceptional efficacy, being hailed as one of the most effective screening methodologies [23].

Krylov’s team has focused extensively on enhancing the efficiency and precision of CE-SELEX by introducing a series of kinetic approaches that integrate binding dynamics into the selection process. They first proposed the nonequilibrium capillary electrophoresis of equilibrium mixtures mode, which allows the simultaneous determination of binding parameters (K_d_, K_on_, and K_off_) and improves selection by separating bound and unbound species from an equilibrium mixture [24]. Building upon this, they developed the equilibrium capillary electrophoresis of equilibrium mixtures, a method that enables the isolation of aptamers with controlled affinities by leveraging differential migration under equilibrium conditions [25]. To further streamline the process, they later introduced the Non-SELEX strategy [26], which eliminates the need for PCR amplification, thereby reducing bias and accelerating selection. In parallel, the methods developed by Qu’s team and other researchers have significantly advanced the field of SELEX for searching for oligonucleotide ligands. Qu’s team has introduced innovative methods such as LpH-CE-SELEX [27]. This method effectively separates protein oligonucleotide complexes from unbound oligonucleotide libraries by inhibiting electroosmotic flow (EOF) in a low-pH environment. The breakthrough of this technology lies in the fact that under low-pH conditions, due to the influence of charge, the complex and unbound library molecules migrate in opposite directions in the capillary, thereby avoiding pollution problems during the separation process and greatly improving the collection efficiency of the complex. In addition, single-step CE-SELEX is another important innovation [28] that integrates mixing, reaction, separation, and detection into a single online step. This one-step online reaction method greatly shortens the experimental time and significantly reduces the required resource consumption. It can adjust the number of complexes faster and make it easier to attain various proportions of target nucleic acid reactions. This approach can enhance sample utilization from 5% to 100%, circumvent waste from multiple incubation injections, and provide a new solution for efficient oligonucleotide screening. Synchronized-competition CE-SELEX further breaks through the limitations of traditional SELEX methods, achieving the ability to compete and select multiple targets simultaneously in a single CE operation. This feature greatly expands the application scope of SELEX technology, providing an efficient and fast solution for the selection of multiple targets in complex samples. Beyond these methods, Qu’s team also explored capillary transient isotachophoresis SELEX, which improves the resolution and enrichment efficiency of aptamer-target complexes by introducing a transient isotachophoresis step, which is especially valuable for challenging targets like cells or low-affinity ligands [29]. Furthermore, fraction-collection CE-SELEX introduced a segmented fraction-collection strategy coupled with RT-PCR quantification to accurately identify and enrich optimal sequences within a single round, effectively reducing selection cycles and minimizing amplification-related contamination [30]. Recently, Wa and colleagues introduced a microbead-assisted CE-SELEX method to improve the collection accuracy of composites [31]. This method utilizes microbeads to immobilize the target, which exhibits significant absorbance changes and migration time differences when bound to single-stranded DNA, allowing for the observation and collection of oligonucleotide aptamer target complexes using ultraviolet indicators. This method significantly improves the sensitivity and accuracy of complex detection and collection. Through this method, oligonucleotide aptamers targeting thrombin were successfully obtained. Additionally, Takao and colleagues introduced a novel method called Microscale Electrophoretic Filtration SELEX [32]. This method partially fills the hydrogel inside the capillary tube, and IgE is injected into it through electrophoresis. Because of the molecular sieve effect, IgE is confined to the hydrogel’s proximal interface, followed by the injection of the DNA library. Subsequently, the unbound single-stranded DNA (ssDNA) is removed by the hydrogel, and the ssDNA with a strong binding ability is eluted and collected. However, compared to traditional CE-SELEX, this apparatus features a larger inner diameter (350 μm) and an extended separation duration (30 min), potentially resulting in increased Joule heating and electrolysis effects.

It is worth noting that CE-SELEX has also been applied to a broader range of targets, including small molecules, cells, and exosomes. To overcome the limited mobility differences between free and bound ssDNA when targeting small molecules, Yang et al. further demonstrated the applicability of CE-SELEX to low-molecular-weight targets by successfully selecting aptamers for clenbuterol hydrochlorid using a capillary zone electrophoresis ultraviolet mode, marking it as one of the smallest targets reported in this field [33]. Qu’s team pioneered the application of capillary electrophoresis for aptamer selection targeting whole cancer cells such as U251 [34]. This approach enabled the precise assessment of ssDNA binding variability across different cell types while simultaneously achieving the efficient separation and quantification of target-bound and unbound sequences within a single CE run, representing a streamlined and effective platform for whole-cell aptamer discovery. Furthermore, the team developed an optimized CE-SELEX strategy tailored for exosome targets, introducing a multi-phase selection process that significantly improved the screening efficiency and binding performance of aptamers toward natural killer-cell-derived exosomes [35].

#### 2.2.2. Cell-SELEX

**Figure 4:**
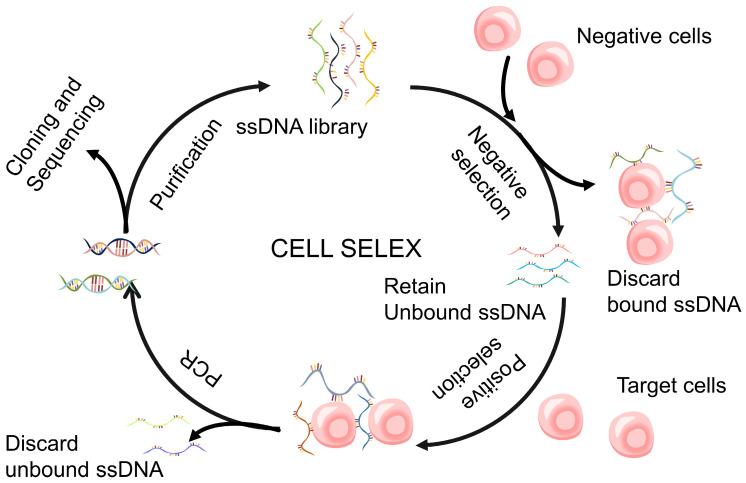
The Cell-SELEX process.

Discovering aptamers that target natural conformational proteins, receptors, and molecular biomarkers present on cell surfaces has become a potentially valuable tool for developing effective disease-specific probes. This method is commonly referred to as Cell-SELEX [36] (Figure 4). The first successful Cell-SELEX trial used a viable pathogenic organism, Trypanosoma Africana Brucella [37]. This study demonstrated the identification of three types of high-affinity RNA aptamers, which are specifically associated with the blood-flow lifecycle stage of parasitic infections. These aptamers were screened and targeted the variant surface glycoprotein protein, which is highly abundant among the peptides on Trypanosoma’s surface. Cell-SELEX technology has multiple advantages in aptamer screening. Firstly, compared to traditional protein SELEX, it does not require prior knowledge of cell biomarkers or protein purification, thus saving time and resources. Secondly, Cell-SELEX provides an opportunity to identify new biomarkers, and successful selection can generate aptamers for unknown biomarkers, which can help discover new therapeutic and diagnostic targets. Most importantly, due to the target molecule being in its natural conformation in Cell-SELEX, the developed aptamers are easier to apply for treatment and diagnosis and do not require the purification or fixation of the target molecule, simplifying the processing process. Therefore, Cell-SELEX has been widely utilized for acquiring highly specific aptamers targeting tumor cells, proteins associated with tumors, virus-infected cells, parasites, and intact cells [38].

In Cell-SELEX, the presence of complex surface components might skew the library towards other highly expressed components. In order to improve selection efficiency and avoid the generation of aptamers targeting biomarkers or molecules on the surface of target cells, the Hicke laboratory has introduced a new variant of the cellular SELEX method called cross-over SELEX, which is used to identify RNA aptamers targeting the tenascin-C protein [39]. In cross-over SELEX, both purified proteins and cells expressing the protein on the surface are used as targets. After several rounds of screening in a study, the enriched sequences in Cell-SELEX pool were further incubated with purified proteins as part of the cross-over SELEX program. After two rounds of cross-over selection, the binding affinity of the enriched aptamer sequences increased by 50 times compared to the cellular SELEX library. Similar to cross-over SELEX, reverse cross-over SELEX is a two-stage selection process entailing the strict selection of recombinant proteins, succeeded by the functional selection of live cells. This technology has been successfully used to discover RNA aptamers that exhibit binding affinity for human transferrin receptors and undergo facile internalization [40]. The Mallikaratchy team proposed the ligand-guided selection method as a variant of cellular SELEX to select specific ligands for known cell surface epitopes. This method utilizes the allocation phases within aptamer selection by introducing pre-selected ligands with high affinity (such as monoclonal antibody mAb) to compete for target epitopes with already bound ligands, thereby achieving the elution of specific ligands. In addition, the Mallikaratchy team also improved the affinity of the aptamer through the manipulation of the reaction system [41], structural modification [42], and dimerization [43], thereby expanding the application scope of this method, which can screen for various cell surface markers. However, it is worth noting that this method has certain limitations when competing with antibodies as high-affinity antibodies targeting the target protein must be obtained first. This limitation may slow down future development and reduce the potential applications of this method. Another variant of Cell-SELEX combines three-dimensional cell culture and Cell-SELEX methods, aiming to develop aptamers targeting cell surface targets. Compared to traditional two-dimensional culture models, three-dimensional cell culture simulates the natural environment for cell growth and development, which may provide a more physiological environment and potentially improve the process of basic research and drug discovery. This cultivation method was developed from a two-dimensional cell culture using magnetic levitation method (MLM) [44]. The study by Souza et al. demonstrated that after nine rounds of selection, eight RNA aptamers were successfully selected for spherical cells formed by invasive prostate cancer cell lines. They cultured prostate tumor cells using the MLM to form a three-dimensional cellular structure [45]. In order to exclude non-specific ligand binding to spherical cells, the study used negative control cells for the first round of selection. The aptamer discovered binds to prostate tumor cells with a nanomolar level of K_d_ and has the potential for application in the treatment and diagnosis of prostate cancer.

#### 2.2.3. Microfluidic and Microarray SELEX

The Microfluid SELEX and Microarray SELEX technologies represent significant advancements in aptamer screening methods, utilizing cutting-edge microfluidic and microarray technologies to significantly optimize the aptamer screening process. The application of these technologies not only greatly improves the throughput and efficiency of screening but also allows for the overall cost of research to be effectively reduced by minimizing the consumption of reagents and samples. Microfluid SELEX improves the repeatability and accuracy of experiments through precise fluid control while Microarray SELEX utilizes high-density aptamer arrays for precise quantitative analysis, further enhancing the accuracy of the screening process. In addition, the rapid iteration ability of these technologies significantly shortens the time cycle from initial screening to final validation, accelerating the development of high-affinity and high-specificity aptamers. Overall, the integrated application of Microfluid and Microarray SELEX technology not only promotes the evolution of aptamer screening methods but also overcomes the disadvantages brought by favorable factors of human operation, greatly improving screening speeds.

Xu’s group has made significant contributions in the field of aptamer screening technology, developing a series of innovative methods to improve screening efficiency and accuracy. They first proposed a protein microarray microfluidic chip technology called PMM-SELEX [46], which immobilizes positive and negative target proteins onto aldehyde-modified microarrays through physical adsorption. These microarrays are then integrated into the microfluidic chip to screen for specific target-protein lactoferrin aptamers. This method achieves online monitoring by sequentially injecting ssDNA libraries into two microarrays containing positive and negative proteins and sequentially implementing negative and positive selection with a fluorescein-labeled library, greatly improving the throughput and accuracy of screening. In addition, the team also introduced a novel method combining Ag10NPs enrichment and SPR imaging [47] to further optimize the detection signal of the SELEX process. Faced with the complexity of microarray channel manufacturing and high equipment costs, Xu’s group continued to innovate and proposed an improved Microarray SELEX platform in 2019 [48]. This platform uses Smart Array and Microarray Scanner without the need to design microfluidic channels, simplifying experimental operations and production processes. By directly fixing the target in microarray form on a glass slide and incubating it with an ssDNA library under static conditions, this method simplifies the binding process between aptamers and targets while maintaining the ability to efficiently screen aptamers.

The Hybarger group first developed a microfluidic SELEX prototype in 2006 [49], which integrates on-chip nucleic acid amplification devices and robot manipulators, achieving automation of the SELEX process. This breakthrough work has laid the foundation for the subsequent development of fully automated microfluidic SELEX systems, which have been optimized in terms of control accuracy and miniaturization. In 2016, Lin’s group developed a microfluidic system that integrated the ssDNA regeneration process [50]. The system uses a microbead-based approach and achieves the coupling of the entire SELEX process through an electrophoretic DNA manipulation scheme. The design of the system includes a heater, a temperature sensor, and a microchannel filled with sol-gel. The effective transmission of the target between the selection room and the amplification room is achieved through the electrophoretic force.

#### 2.2.4. Integration of NGS into SELEX Protocols

In traditional SELEX, the enriched library is typically cloned and analyzed using classical Sanger sequencing to determine the sequences of the individual aptamer. However, with the development of NGS technologies, it has become increasingly common to integrate sequencing and computational analysis into various SELEX protocols. Instead of analyzing only a few aptamer candidates, NGS enables the real-time monitoring of aptamer enrichment dynamics across multiple selection rounds. This facilitates the early identification of potential binders, potentially reducing the number of selection cycles required. By sequencing enriched pools at each round, researchers can perform global analyses of motif enrichment and identify large sets of candidate aptamers simultaneously. To fully leverage the extensive data generated by NGS, appropriate computational tools are essential for classifying sequences and selecting aptamers with desirable characteristics. This integration is especially valuable when designing aptamers for complex therapeutic applications, such as those that bind to specific targets and actively modulate biological functions—e.g., aptamers that regulate anticoagulant levels in real time [51].

The integration of NGS with SELEX has greatly enhanced the exploration of nucleic acid–protein interactions. For example, Lorentz et al. applied a genome-derived RNA library to investigate the binding specificity of the E. coli RNA-binding protein Hfq. By coupling SELEX with high-throughput sequencing, they identified Hfq-associated RNA motifs using tools like MEME and mapped them across the bacterial genome [52,53]. Enrichment patterns were evaluated through comparative analysis with neutral control datasets, enabling the discovery of novel RNA targets and verification of strong binding affinities in the nanomolar range. The results also suggested a regulatory mechanism involving Hfq’s interactions with antisense RNAs. This strategy has also been employed to study other transcriptional regulators, such as PapX in *E. coli* [54], revealing its target sequences and functional pathways [55]. Bioinformatics platforms such as TFAST have been developed to enhance the processing of extensive sequencing datasets. These platforms provide automated analysis pipelines that significantly improve both accuracy and reproducibility.

Although NGS is not part of the selection process, its integration as a downstream analytical tool allows the quantitative monitoring of sequence enrichment and diversity. These advances position NGS-enhanced SELEX as a powerful and scalable framework for decoding complex biomolecular interactions.

### 2.3. Challenges and Improvement Strategies for SELEX Technology

Despite significant advancements in SELEX technology over the past three decades, its widespread adoption and effectiveness in research and applications continue to be hindered by certain limitations.

Firstly, the development of SELEX technology is limited by challenges such as the difficulty in identifying and obtaining suitable target molecules. SELEX relies on the identification of specific target molecules, which are essential for initiating the selection process using a random aptamer library. However, in many cases, particularly within complex biological systems, pinpointing these specific targets can be challenging. This uncertainty limits the applicability of SELEX in certain biological contexts as the absence of clearly defined targets can result in aptamers being unable to bind effectively. Secondly, striking a balance between affinity and specificity poses a significant challenge during the SELEX process. While SELEX technology can yield aptamers with high affinity, there are instances where these aptamers lack the necessary specificity, resulting in unintended binding with non-target molecules [56,57]. This compromises the accuracy and reliability of aptamers, particularly in scenarios involving the identification and isolation of target molecules within complex samples. Additionally, accurately predicting the structures of selected aptamer sequences from SELEX remains a formidable challenge. Although bioinformatics methods can be employed to predict aptamer secondary structures, the complexity of sequences and structural diversity can lead to inaccuracies in prediction, thereby impeding the understanding and application of aptamer properties.

To overcome these limitations of SELEX technology, future research can explore several avenues for improvement. Firstly, efforts can be directed towards developing a broader range of SELEX variants capable of accommodating different types of targets and samples. For instance, reverse SELEX technology offers the potential to directly screen aptamers from complex samples without the need for pre-defined targets, thereby expanding the applicability of SELEX to more intricate biological systems. Secondly, the optimization of screening methods through the integration of machine learning and artificial intelligence technologies holds promise. By establishing models to predict aptamer–target binding affinity and specificity, the screening processes can be more effectively guided, leading to enhanced aptamer performance. This approach leverages the strengths of both computational and experimental methods and can potentially expedite the discovery and optimization of aptamers. For example, Douaki’s group integrated techniques from machine learning and bioinformatics to devise customized methods for designing aptamers targeting ammonium ions (NH_4_^+^) in water [58]. This approach involved utilizing deep neural network algorithms to compile a dataset of aptamers with a molecular weight below 900.0 g/mol from the literature, followed by model training. Subsequently, the research team constructed a candidate library comprising 108 entries, each consisting of 27 bases, and employed machine learning models to forecast the binding affinity of individual sequences with the target. Finally, through molecular docking simulations and affinity assays, aptamers exhibiting high binding affinity to ammonium ions (K_d_ 6.6 mM) were selected. Lastly, there is a need to bolster the development of predictive and analytical tools for aptamer structure and function. The integration of diverse bioinformatics methods and experimental validation can enhance understanding of aptamer structure and function, guiding their design and application. This encompasses the development of more accurate sequence analysis and structural prediction tools alongside the establishment of extensive databases for storing and sharing aptamer information.

In conclusion, while SELEX technology has made significant strides in nucleic acid aptamer research, it still grapples with limitations that impede its practical application and efficiency. Future research endeavors should focus on surmounting these limitations through the development of innovative technological approaches and the integration of multidisciplinary research methodologies, thereby advancing nucleic acid aptamer research and its applications.

## 3. Application of Aptamers in Therapeutics

Since 1990, researchers have focused on harnessing the flexibility of aptamers as therapeutic agents, enabling their customization, reversibility, and utility in diagnosing and treating diseases. Therapeutic aptamers currently undergoing clinical trials primarily exert their effects by inhibiting the activity of pathological target proteins.

### 3.1. Eye Disorders

#### 3.1.1. Vascular Endothelial Growth Factor

**Figure 5:**
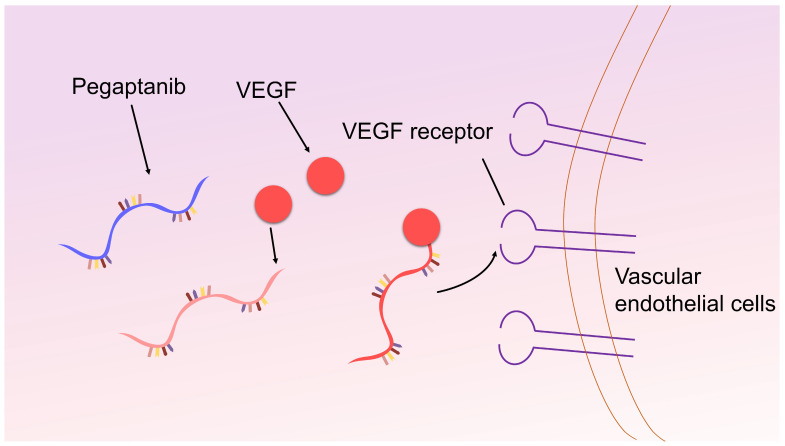
Pegaptanib is a 28-nucleotide RNA aptamer that specifically binds to VEGF, thereby inhibiting endothelial cell proliferation, tissue-level vascular leakage, and cardiovascular formation.

In recent years, there have been significant advancements in the treatment of macular degeneration. Firstly, neovascularization is a pathological process associated with age-related macular degeneration (AMD), the retinopathy of prematurity, and diabetic retinopathy [59,60]. The upregulation of vascular endothelial growth factor (VEGF) is considered a common characteristic in these diseases, thus prompting attention towards VEGF-targeted treatment strategies. Pegaptanib, formerly known as NX-1838 [61], has emerged as a noteworthy aptamer drug in AMD therapy (Figure 5). With a high affinity for VEGF165, pegaptanib effectively inhibits VEGF-mediated capillary leakage by binding to a 40 kDa polyethylene glycol (PEG) molecule, thereby laying the groundwork for intraocular anti-angiogenic therapy. Clinical trial findings have demonstrated the significant efficacy of pegaptanib both as a standalone treatment for AMD patients and in combination with photodynamic therapy. However, the drug is accompanied by certain side effects, including endophthalmitis, elevated intraocular pressure, and rare instances of allergic reactions and similar hypersensitivity reactions [62].

As research progresses, aside from pegaptanib, attention has also been drawn to two other targets. Firstly, Pegpleranib, a platelet-derived growth factor DNA aptamer, has shown potential for improving vision in AMD treatment when used in combination with ranibizumab. The second target is complement component C5, for which the aptamer drug Avacincaptad pegol, combined with ranibizumab, has also demonstrated vision improvement. The onset of AMD is influenced by various factors, including genetics and environmental elements, often involving the accumulation of undegraded waste around the retinal pigment epithelium. AMD manifests in two primary forms: wet and dry. Treatment for wet AMD primarily revolves around inhibiting neovascularization while dry AMD poses greater challenges in management. Current research efforts predominantly focus on drug design utilizing oligonucleotide aptamers. Hence, ongoing research delves into exploring diverse targets and treatment modalities tailored for different types of AMD [63].

#### 3.1.2. Infections of the Eye

Eyes are susceptible to various infections, particularly those caused by viruses. Gene therapy based on oligonucleotides has emerged as a crucial treatment method for combating viral eye infections, given that the progression of viral diseases hinges on the interaction between viral genes and the host genome.

Common viral eye infections encompass the herpes simplex virus (HSV), coxsackievirus A24, varicella-zoster virus (VZV), and cytomegaloviru. Alongside commercially available antiviral drugs, antisense therapy stands out as a potent treatment avenue for these infections. Acute retinal necrosis is a condition affecting immunocompromised individuals resulting from HSV-1 and VZV infections. Targeting tumor necrosis factor (TNF) α with antisense oligonucleotides has demonstrated significant efficacy in combating this infection [64]. Additionally, phosphodiester morpholino oligomers tailored to the HSV-1 gene have exhibited promising therapeutic effects [65].

Herpetic stromal keratitis, induced by HSV-1, represents an immune-mediated corneal disease leading to blindness. In this context, antisense therapy directed against TNF α or interferon γ has shown effective recovery outcomes [66,67]. These research findings and treatment modalities offer novel insights into addressing eye infections, highlighting the potential application value of gene therapy in ophthalmology.

### 3.2. Thrombosis Disease

The development of antithrombotic aptamers entails leveraging specific extracellular targets and designing oligonucleotides with antiviral properties to address potential bleeding events. The crux of this strategy hinges on selecting suitable targets to ensure the accurate recognition and binding of the aptamer to the target molecule. The design of these aptamers aims to mitigate the risk of bleeding while preventing thrombosis. Through research and development of these aptamers, novel treatment options can be offered for thrombotic diseases, potentially reducing adverse reactions and complications associated with traditional anticoagulant therapy.

#### 3.2.1. Thrombin

Thrombin serves as a pivotal coagulation protease crucial for the blood coagulation process. It originates from the activation of prothrombin by thromboplastin during thrombus formation in the coagulation cascade. Once formed, thrombin engages in various facets of thrombosis through diverse pathways. Among its primary functions is the conversion of fibrinogen into fibrin, thereby facilitating blood clot formation. This process enhances clot structural stability by establishing a fibrin network, which aids in hemostasis. Furthermore, thrombin can activate additional coagulation factors such as coagulation factor V and coagulation factor VIII, establishing a positive feedback loop that expedites thrombus formation. In the realm of disease treatment, thrombin emerges as a pivotal drug target. Anticoagulant medications like warfarin and heparin inhibit thrombin activity to prevent and manage thrombosis [68,69]. Thus, as a critical coagulation factor, thrombin plays an indispensable role in both physiological and pathological processes. A comprehensive understanding of its functionality holds promise for the development of more effective treatment strategies to combat the challenges posed by thrombotic and inflammation-related diseases.

Beyond the indirect inhibition of thrombin within the coagulation pathway, aptamers are capable of directly binding to thrombin and rendering it inactive, demonstrating high specificity and potent inhibitory efficiency. The seminal work by Bock et al. in 1992 introduced a DNA aptamer targeting thrombin, marking one of the first instances of DNA-based aptamer selection and among the earliest aptamers developed for a coagulation protein [70]. Subsequent research has focused on the clinical translation of thrombin aptamers [71]. Among them, HD1 and NU172 are two representative thrombin-specific DNA aptamers that have progressed into clinical evaluation due to their high binding affinity and anticoagulant potential. HD1, a 15-nucleotide aptamer, targets thrombin’s exosite I and has demonstrated the ability to inhibit thrombin-mediated fibrin formation. Despite achieving rapid anticoagulation in early clinical trials, its short half-life and high dosing requirements have limited further development. Modifications aimed at enhancing HD1’s stability and bioactivity, such as nucleotide substitutions and structural alterations, have shown varying degrees of success [72,73]. NU172, on the other hand, is an unmodified DNA aptamer with stronger anticoagulant activity than HD1, though it also suffers from rapid clearance in vivo [31]. Recent efforts have focused on improving its pharmacokinetic profile through chemical modifications [74]. Both aptamers exemplify the challenges and opportunities in translating nucleic acid therapeutics into clinical anticoagulants.

#### 3.2.2. von Willebrand Factor

**Figure 6:**
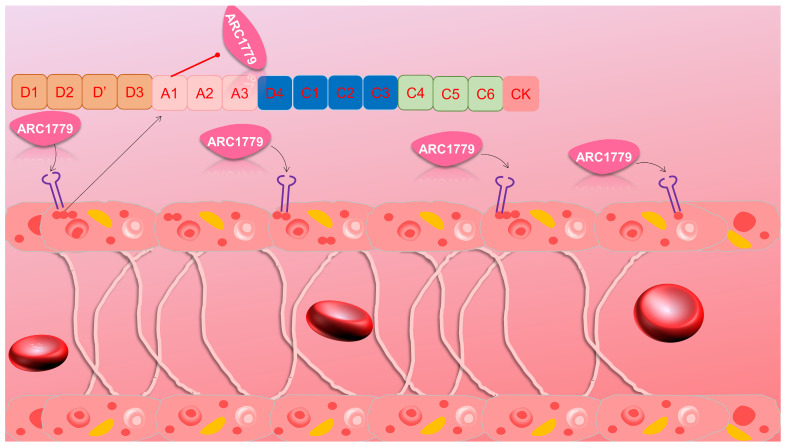
ARC1779 is a 40nt DNA/RNA nucleic acid aptamer that can bind to the A1 region of human vWF. By utilizing the vWF mediated activation pathway, it can block pathological thrombosis and treat vascular hemophilia.

During the blood coagulation process, von Willebrand factor (vWF) becomes exposed on the surface of blood vessels following endothelial injury, facilitating platelet adhesion. ARC1779, a DNA/RNA aptamer consisting of 49 nucleotides, selectively binds to the A1 domain of vWF, disrupting the interaction between vWF and the platelet receptor GPIb (Figure 6). Consequently, this inhibits platelet activation and mitigates the potential for pathological thrombosis [75,76]. ARC1779 exhibits promising therapeutic potential in the treatment of von Willebrand disease and acute coronary syndrome [77].

Clinical studies have validated the efficacy of ARC1779. A controlled trial demonstrated that ARC1779 exerts a dose-dependent and concentration-dependent inhibitory effect on vWF activity and platelet function. Moreover, it is well tolerated within the therapeutic dose range without significant adverse events. Another study illustrated that ARC1779 can completely inhibit the activity of the A1 domain of vWF, preventing desmopressin-induced thrombocytopenia in patients with von Willebrand disease type 2B. Apart from ARC1779, other aptamers targeting vWF, such as ARC1772 and a novel RNA aptamer, have shown potential therapeutic effects. These effects have been corroborated in animal experiments demonstrating their ability to inhibit platelet aggregation. To mitigate bleeding risks, some studies have also developed antidote oligonucleotides capable of rapidly reversing the activity of aptamers.

Recent studies have highlighted the potential of BT200, a pegylated RNA aptamer targeting the A1 domain of vWF, in modulating both platelet function and vWF metabolism. In the context of large artery atherosclerosis (LAA) stroke, BT200 has demonstrated efficacy in inhibiting vWF-mediated platelet activity in patient-derived blood samples. Notably, its functional performance remains unaffected by conventional antiplatelet agents, suggesting a promising adjunctive role in secondary stroke prevention for LAA patients [78].

Beyond its antiplatelet activity, BT200 also exhibits a capacity to increase circulating vWF and factor VIII levels. This effect appears to be linked to its interference with the interaction between vWF and clearance receptors, particularly those on macrophages. Mechanistic investigations revealed that BT200 binding near a conserved lysine cluster (K1405–K1408) on the A1 domain of vWF could significantly reduce recognition and uptake by lipoprotein receptor-related protein 1 and other macrophage-associated scavenger receptors in a study. Mutagenesis studies further confirmed the importance of this lysine-rich region in receptor-mediated clearance as its alteration resulted in a prolonged vWF plasma half-life [79]. These findings collectively support BT200 not only as a modulator of platelet- vWF interaction but also as a novel agent capable of regulating vWF homeostasis via the inhibition of its degradation pathways.

Alongside ARC1779 and BT200, other aptamers, such as DTRI-031, have also been explored for their potential in modulating platelet function and preventing thrombosis. DTRI-031, by disrupting the interaction between vWF and platelets, effectively prevents thrombus formation. In preclinical models, this aptamer has shown significant anti-thrombotic activity, including the inhibition of thrombosis in a murine carotid artery injury model and a reduction in platelet aggregation in whole blood samples [71,80].

#### 3.2.3. Coagulation Factor Ixa

Coagulation factor IXa (FIXa) is a key component of the intrinsic coagulation pathway. Upon activation, FIXa binds to its cofactor VIIIa (FVIIIa) on phospholipid surfaces to form a complex, which catalyzes the conversion of factor X to Xa. This process markedly enhances thrombin generation, thereby contributing to thrombus formation. Given its central role in coagulation, FIXa has emerged as a critical target for the development of anticoagulant therapies [81,82]. Currently, RNA aptamers are among the main strategies being explored for FIXa inhibition, with REG1 representing a prominent example [83].

REG1 comprises the anticoagulant RB006 and its complementary reversal agent RB007. RB006 disrupts thrombin formation by specifically binding to and inhibiting the activity of FIXa. RB007 acts as a detoxifying agent, swiftly and safely neutralizing the anticoagulant effect of RB006 [84]. FIXa plays a pivotal role in the coagulation cascade, rendering it a potential and promising target for therapeutic research [85].

The REG1 anticoagulant system has undergone clinical evaluation for acute coronary syndrome (ACS) and coronary artery disease [86,87]. The RADAR trial, a partially blinded study, assessed the effects of aptamers/antidotes on patients with ACS and undergoing percutaneous coronary intervention (PCI) [86]. Beyond assessing whether the aptamer can function as an independent anticoagulant for ACS and PCI, the study also evaluated the percentage of antidote reversal of the aptamer. Notably, the aptamer provided sufficient anticoagulant effects in both clinical scenarios.

### 3.3. Viral and Neurological Diseases

Aptamers have recently gained attention for their application in non-traditional therapeutic areas, particularly in infectious and neurological conditions.

Following the emergence of the severe-acute-respiratory-syndrome coronavirus, numerous studies have explored aptamers capable of binding to viral targets such as the spike glycoprotein and the RNA-dependent RNA polymerase. Notably, several aptamers have demonstrated strong binding affinity for the receptor-binding domain of the spike protein, thereby interrupting the viral attachment process by blocking the interaction with the host angiotensin-converting enzyme 2 receptor. In addition to their direct antiviral effects, aptamers have also been evaluated as targeted delivery vectors for therapeutic agents with the aim of improving drug localization and minimizing systemic exposure [88,89].

In the treatment of neurodegenerative disorders, aptamers have demonstrated considerable potential by selectively targeting pathological protein aggregates. In Alzheimer’s disease, for instance, aptamers that bind to oligomeric forms of amyloid-beta or hyperphosphorylated tau have been shown to inhibit fibril formation and reduce neurotoxicity [90,91]. Similar strategies have been applied to Parkinson’s disease, where aptamers targeting misfolded alpha-synuclein aim to prevent the spread of toxic aggregates and support their clearance [92,93,94]. These nucleic acid ligands not only offer high target specificity but may also be employed to modulate neuroinflammation and improve disease outcomes through precise molecular intervention.

The human papillomavirus (HPV), particularly high-risk genotypes such as HPV16 and HPV18, plays a key role in the development of cervical and other epithelial cancers. Aptamer-based therapeutics have focused on interfering with the viral oncogenes E6 and E7, which are responsible for degrading host tumor suppressor proteins like p53 and Rb. By disrupting these interactions, aptamers may help restore normal cell cycle regulation and suppress tumorigenesis. Furthermore, aptamers have been utilized as delivery systems for small interfering RNAs or chemotherapeutic agents, thereby enhancing intracellular uptake and selectivity toward HPV-infected cells [95,96].

### 3.4. Cancer

Aptamers theoretically possess specific recognition abilities for almost all cancer proteins, cancer metabolites, and cancer cells. This makes aptamers highly anticipated tools in the field of cancer diagnosis, especially for those lacking antibodies or unable to find the required selective biomarkers [17].

Aptamers have shown potential for development in the field of biosensing, and related research is growing exponentially. Some aptamer-based models exhibit extremely high sensitivity, especially in detecting cancer cells, with enormous potential. By using magnetic relaxation switch sensing with magnetic nanoparticle (MNP) cluster sizes, even in 250 µL real biological samples, the presence of cancer cells can be successfully detected by combining aptamer–MNP complexes of 10 cancer cells [97]. The combination of aptamers and nucleic acid amplification platforms further improves the sensitivity of detection [98]. For example, using aptamers to amplify HL-60 cells after binding can achieve a precise quantitative analysis of cancer cells. The aptamer exhibits effective enrichment ability towards circulating tumor cells, especially in microfluidic devices [99]. By implementing a dense layer of aptamers on a nanostructured substrate, the matching efficiency for overexpressed target proteins on the cell membrane has been improved. Microchannels with different structures, such as with a small height, low flow velocity, and chaotic flow patterns, can increase the probability of cell binding. Electrochemical detection methods have shown excellent sensitivity in cancer cell detection. The latest research reports on TLS11a aptamer-modified gold disk electrodes, which successfully detected as few as two HepG2 cancer cells in 1 mL of cell suspension using electrochemical impedance spectroscopy [100]. This advancement demonstrates the potential of aptamer-based technologies in enhancing the sensitivity and specificity of cancer detection methods. Another study showed that single MEAR cancer cells in a sample of 109 whole blood cells were successfully detected using a glassy carbon electrode modified with dual aptamers TLS1c and TLS11a [101]. This method demonstrated higher detection sensitivity compared to single-aptamer modification methods. In the detection of cancer protein biomarkers, aptamer-based methods have lowered the detection limit to easily reach the target protein concentration within the range of picomoles. A study even suggests that using aptamer-conjugated gold nanoparticles [102], unlabeled target proteins can be electrochemically detected at five femolar concentrations. Single-molecule target protein detection has also been achieved using chemically modified graphene field-effect transistors. These transistors capture and isolate cancer cells at the single-cell level through signal amplification, providing a deeper understanding for cancer diagnosis research.

Although aptamer-based methods have shown excellent performance in laboratory environments, further validation is still needed in clinical applications. Aptamer-based detection and isolation methods still face challenges in real biological samples due to the high enrichment of non-target blood proteins in most proteomic methods. Additionally, the heterogeneity of cancer cells and the diversity of related proteins add to these difficulties [103]. Therefore, before applying these methods to clinical practice, it is necessary to fully validate them. Meanwhile, in order to achieve a more accurate diagnosis, it is necessary to carefully select the aptamer, considering its affinity and interaction characteristics with the target protein [17]. Ultimately, understanding the biochemical properties of the aptamer, such as its K_d_ value, conformational state, buffering conditions, and temperature, is crucial for ensuring its performance [104]. In this paper, we have summarized representative aptamers based on their types, targets, stages, applications, and modifications, as shown in Table 2.

### 3.5. Challenges in Aptamer-Based Therapeutics

Although selecting aptamers as therapeutic applications is relatively simple, their challenges in adapting to the in vivo environment require extensive research (Table 3). Compared with traditional protein therapies such as therapeutic antibodies, aptamers face different pharmacokinetic issues due to their larger molecular weight and difficulty in crossing biological barriers such as cell membranes. These include but are not limited to the degradation by serum nucleases, rapid renal filtration, and non-specific immune activation, all of which severely limit the in vivo therapeutic efficacy of the aptamer [108]. To address these issues, various chemical modification and coupling techniques have been developed to improve the pharmacokinetic properties of aptamer-based therapeutic drugs, thereby enhancing their stability and therapeutic efficacy in vivo [109].

#### 3.5.1. Aptamer Stability

Humanized antibodies exhibit significant advantages over other drug entities in terms of circulating half-life, ranging from several days to several weeks [110]. In contrast, unmodified nucleotides may only have a half-life of a few minutes in serum [111]. This unfavorable pharmacological characteristic is one of the key challenges that constrain the practical clinical application of oligonucleotides. Two main factors contribute to this: oligonucleotides are easily degraded by nucleases and are excreted in the kidneys. Biological fluids contain abundant nucleases, including exonucleases and endonucleases, which can cleave the phosphodiester bonds of single- and double-stranded oligonucleotides. The degradation time of oligonucleotides in the blood depends on their structure, ranging from a few minutes to tens of minutes [112]. Due to this short half-life, which is not suitable for therapeutic applications, it is usually necessary to chemically modify oligonucleotides to improve their stability in serum [113].

In clinical research, most aptamers undergo chemical modifications to enhance their resistance to nucleases and to increase their binding affinity simultaneously. So far, innovations including 2′-aminopyridines, 2′-fluoropyridines, and modifications like 2′-O-methyl nucleotides and locked nucleic acids have been effectively integrated within the SELEX process to enhance its efficacy [114,115,116,117,118]. Additionally, SomaLogic has innovated with the introduction of SOMAmer technology, which stands for Slow Off-rate Modified Aptamer selection [119]. This method uses modified deoxyuridine with hydrophobic functional groups at the C5 position, exhibiting good DNA polymerase tolerance. L-RNA and L-DNA oligonucleotides belong to a special type of oligonucleotides known as “spiegelmers” (mirror oligonucleotides). Nucleases in human blood and serum cannot recognize these mirror oligonucleotides because their natural D-sugar motifs lack recognition by nucleases, which have specificity for D-sugar motifs. The preparation of mirror oligonucleotides utilizes the principle of mirror symmetry to selectively target desired targets by generating enantiomeric images of natural D-RNA oligonucleotides. Subsequently, after selection, the required L-RNA sequences are synthesized using L-RNA phosphoramidites for chemical synthesis to bind to natural targets [120]. In clinical trials, NOX-A12 has demonstrated efficacy against various solid tumors and multiple myeloma. Additionally, studies by Joyce et al. have demonstrated that the selected L-RNA oligonucleotides can inhibit specific microRNA processing [107]. Given that L-RNA is incapable of base-pairing with D-RNA, these oligonucleotides operate via precise three-dimensional conformations, eschewing reliance on traditional Watson–Crick base complementarity.

The continuous advancement of chemical modifications is pivotal for enhancing the stability of aptamers in vivo. Beyond the conventional 2′-substitutions and locked nucleic acids, innovative modifications can be engineered to not only resist nuclease degradation but also optimize pharmacokinetics and tissue penetration. Furthermore, the application of computational methods and machine learning algorithms can significantly facilitate the rational design of these modifications. By predicting their impact on stability and binding affinity, these advanced computational approaches can accelerate the discovery and optimization process.

#### 3.5.2. Renal Filtration

The molecular weight of most aptamers typically ranges from 5 to 15 kDa (equivalent to a length of 15–50 nucleotides), with an average diameter of less than 5 nm [121]. Although these aptamers may exhibit good resistance to nuclease-mediated degradation, their small molecular size makes them susceptible to glomerular filtration. A common solution to overcome the problem of rapid excretion by the kidneys is to conjugate them with high-molecular-weight groups (such as PEG [122], cholesterol [123,124], proteins [125], polymers [126,127], or nanomaterials [128,129]) to form multivalent molecules exceeding the glomerular filtration threshold (30–50 kDa) to prevent renal filtration. PEG is a hydrophilic biocompatible material approved by the US Food and Drug Administration (FDA) [130] that has been widely used in 12 human biopharmaceuticals [131]. PEG has various commercial sizes (ranging from 0.3 to 10,000 kDa) and different end-functional groups for chemical coupling. For aptamer applications, PEG is widely used to increase their size and improve their residence time in circulation to enhance their stealth effect [132]. After the PEGylation of Macugen, its half-life in plasma was correspondingly increased to 9.3 h and 12 h through intravenous or subcutaneous injection. More impressively, when introduced into vitreous fluid, its half-life was extended to an extraordinary 94 h [61,133].

The coupling between proteins and PEG is usually carried out through random reactions of lysine side chains, which often leads to product mixing and the loss of activity. In contrast, aptamers, due to their chemical synthesis and devoid of multiple functional groups commonly found in proteins, can be site-specifically modified by introducing a single functional group (such as alkylamine). This single functional group can serve as an exclusive site for coupling with other molecules without damaging the structure or function of the aptamer. The site-specific conjugation point is strategically placed to allow for versatility in attachment, ensuring that the addition of PEG does not interfere with the functional activity of the resulting conjugate. Compared to single aptamers, well-designed multivalent aptamers exhibit superior comprehensive performance in terms of binding affinity, specificity, biological efficacy, and circulation time. For instance, tetramer aptamer conjugates exhibit higher retention rates in circulation while improving pharmacokinetic characteristics.

To counteract rapid renal clearance, the development of modular aptamer architectures, such as aptamer–drug conjugates, aptamer–protein chimeras, or aptamer–nanoparticle hybrids, could be pursued with a focus on tunable size, charge, and biodegradability. These complexes, tailored to surpass renal filtration thresholds, could incorporate advanced release mechanisms triggered by specific physiological conditions or enzymatic actions at the target site. Additionally, the exploration of biomimetic or bioinspired coatings that mimic endogenous molecules could enhance the circulatory lifespan of aptamers while maintaining their biocompatibility.

#### 3.5.3. Toxicity

Multiple factors, such as the ligand sequence, administration pathway, and dosage (including single and cumulative doses), can all contribute to adverse reactions [134]. Aptamers administered via intravenous injection are generally considered to have good tolerance. However, there are still reports of dose- and sequence-dependent adverse reactions [135]. Research indicates that intravenously injected aptamers can be rapidly distributed to tissues in multiple organs, particularly showing significant accumulation in the liver, kidneys, and spleen. The qualitative evaluation of oligonucleotide accumulation in these tissues has been conducted using immunohistochemistry, in situ hybridization techniques, or histopathological analysis.

Geary et al.’s study demonstrated the accumulation of antisense aptamers targeting human TNFα in the kidneys, liver, lymph nodes, and spleen in mice and primates [136]. When administered via the peritoneal route (at a dose of 100 mg/kg) and intravenous route (at a dose of 50 mg/kg), reversible abnormalities were observed in the kidneys and liver of mice, which returned to normal within 4 to 13 weeks [137,138]. The study also noted monocyte infiltration and Kupffer cell hypertrophy in the liver, along with basophilic granules in the cytoplasm of renal tubular epithelial cells [139,140]. ISIS 2302, a 20-mer antisense phosphorothioate oligonucleotide targeting human intercellular adhesion molecule-1 mRNA, unexpectedly exhibited anticoagulant effects [141]. In vitro experiments showed that when the ISIS 2302 concentration exceeded 100 µg/mL, it prolonged the prothrombin time and thrombin time in human plasma. Furthermore, a placebo-controlled trial evaluating ISIS 2302’s efficacy in treating Crohn’s disease revealed that the intravenous infusion of 2 mg/kg ISIS 2302 for over 2 h transiently increased activated the partial thromboplastin time by approximately 10 s within 2 to 4 h post administration [142]. However, no prolonged bleeding time was observed in the study.

The activation of the innate immune response is mediated by Toll-like receptors (TLRs) 3, 7, 8, and 9. TLR3 specifically recognizes double-stranded RNA, while TLR7 and TLR8 are sensitive to single-stranded RNA, and TLR9 identifies unmethylated CG motifs (CpG motifs) in DNA sequences. Given the susceptibility of unmodified RNA to intracellular nucleases and the ability of 2′-modification to effectively block TLR response [143], investigating TLR9’s role becomes crucial in exploring the potential adverse effects of therapeutic ligands. However, when analyzing in vivo responses to small interfering RNA (siRNA), other types of TLRs must not be disregarded. TLR9 activation promotes antigen-specific B cell activation and the release of cytokines including interleukin-6 and interferon [144]. Consequently, immune responses orchestrated by TLR9 are being considered as a potential therapeutic approach, especially in scenarios like tumor treatment that necessitate immune activation [145].

Significantly, even with the intravenous injection of pegaptanib up to 1000 times the clinical dose in primates, no significant immunogenicity or restrictions were observed in a study [146]. While antibody development for synthesizing oligonucleotides is uncommon [147], it is worth noting that PEG antibodies may induce adverse reactions due to frequent exposure to polyethylene glycol products [148]. Therefore, adopting cautious formulations or administration procedures is crucial for advancing adaptive therapy.

Strategies to minimize off-target effects and immune responses are crucial for the effective therapeutic application of aptamers. Precision medicine approaches that utilize patient-specific or disease-specific biomarkers can guide the development of aptamers with enhanced specificity. This can help reduce their accumulation in non-target organs and mitigate adverse reactions. Additionally, understanding the interactions between aptamers and innate immune receptors, particularly TLRs, can inform the design of immunosilent or immunomodulatory aptamers. These specially designed aptamers can either evade the immune system or strategically engage it for therapeutic benefit. Furthermore, employing novel delivery vehicles to shield aptamers from unintended interactions until they reach their target site can further reduce toxicity and enhance therapeutic efficacy.

## 4. Application of Aptamers in Drug Delivery

Modern medicine aims for targeted therapies, but many drugs lack selectivity, causing off-target side effects. Aptamers, which can serve both as therapeutic agents and drug delivery vehicles, offer a solution. These nucleic acid molecules bind specifically to target molecules, directing drugs precisely to diseased sites and enhancing drug stability. Aptamers improve drug efficacy and reduce side effects compared to conventional methods like chemotherapy and radiation therapy. They can be combined with various therapeutic agents, including siRNA, miRNAs, reverse-transcription factors, chemotherapy drugs, toxins, and nanocarriers. This precise targeting increases local drug concentrations and therapeutic effectiveness. This approach opens new possibilities for personalized medicine and advanced therapeutic modalities.

### 4.1. Targeted Drug Delivery

#### 4.1.1. Prostate-Specific Membrane Antigen (PSMA)

Prostate-specific membrane antigen (PSMA) is a membrane-binding glycoprotein highly localized to prostate epithelial cells. The increased expression of PSMA is associated with prostate cancer, particularly in hormone-refractory diseases. Given its membrane-binding properties, PSMA serves as an ideal sentinel molecule for targeting prostate cancer cells [149]. PSMA has been successfully combined with various therapeutic oligonucleotides through covalent fusion or physical assembly to achieve targeted delivery [150]. A9 and A10 are 2′-fluoro-2′-deoxypyrimidine (2′-F-Py) RNA aptamers selected for binding to the extracellular domain of PSMA using immobilized proteins. The use of 2′-F-Py not only greatly enhances RNA resistance to abundant serum nucleases [151] but also enhances RNA thermal stability. In many subsequent studies, PSMA-specific aptamers have been utilized to deliver small molecule therapies and siRNA covalently or noncovalently bound to the aptamers [152]. PSMA aptamers can attach to therapeutic oligonucleotides via molecular platforms or noncovalent bridging strategies. For instance, two biotinylated PSMA aptamers with biotinylated siRNA have been physically assembled onto a streptavidin connector. This assembly produces multivalent conjugates that selectively deliver siRNA to PSMA-positive cells, thereby triggering specific RNA activity [153].

#### 4.1.2. Nucleolin

Nucleolin is a protein that is overexpressed on the cell membrane in many tumors [154]. It can serve as a binding protein for several ligands related to angiogenesis and tumorigenesis. Nucleolin is present in the cytoplasm, nucleoplasm, and nucleolus and is exploited by selected pathogens to enter cells. AS1411 is an oligonucleotide aptamer rich in guanosine, which can bind to nucleolin and internalize in tumor cells. AS1411 exhibits good tolerability at therapeutic doses, targeting tumor cells overexpressing nucleolin and demonstrating good safety profiles. It has entered phase-II clinical trials for acute myeloid leukemia and renal cell carcinoma [155], showing mild-to-moderate side effects in recurrent acute myeloid leukemia and renal cell carcinoma. The promising potential of AS1411 lies in its ability to conjugate with drugs and nanoparticles. When combined with AS1411, the drug will target tumor cells, achieving targeted therapy with fewer systemic side effects compared to traditional methods. AS1411 can also bind to nanoparticles capable of detecting nucleolin [156], with concentrations far lower than those currently used in laboratory technologies for cancer diagnosis. AS1411 holds broad potential in revolutionizing cancer diagnosis and treatment [157].

#### 4.1.3. HIV

Another significant application of aptamer-mediated drug delivery pertains to formulating strategies to combat HIV. Within HIV-infected individuals, the envelope glycoprotein gp120, found on the exterior surface of viral particles, engages with a cluster of differentiation 4 (CD4) receptors and C-C chemokine receptor 5 (CCR5) situated on the host cell’s exterior, thereby enabling the virus’s infiltration into immune cells [158]. Given the pivotal role of this interaction in virus invasion, individuals with particular CCR5 mutations may exhibit immunity to certain strains of HIV [159]. Nonetheless, such mutations are uncommon. Hence, drugs that disrupt the binding process can offer potential therapeutic avenues. The groundbreaking research by Rossi and colleagues has resulted in the advancement of gp120 aptamers, which serve a dual function: firstly, inhibiting the binding between gp120 and CD4 receptors, and secondly, functioning as siRNA delivery agents specific to particular cell types [160]. Gp120 RNA aptamers specifically transport anti-HIV siRNA to cells infected with HIV-1 and suppress HIV-1 activity in vitro [161].

Subsequent studies conducted in humanized mouse models have demonstrated that systemically administered gp120 aptamer–drug conjugates can significantly suppress HIV replication, with inhibitory effects reaching several orders of magnitude. This suggests that aptamer–drug conjugates offer a promising therapeutic approach for HIV infection.

### 4.2. Aptamer–Drug Conjugates

#### 4.2.1. Covalent Conjugation

The covalent linkage between nucleic acid aptamers and chemotherapy drugs represents a promising strategy for targeted drug delivery. Huang et al. first proposed, in 2009, the concept of an aptamer–drug conjugate (ApDC), where DNA aptamer sgc8 is covalently linked to doxorubicin (Dox), enabling controlled drug release in acidic environments and enhancing selectivity towards cancer cells [162]. However, there was an issue of limited drug binding observed during experimental procedures. To overcome this limitation, Boyaciogul et al. introduced an innovative approach involving the synthesis of dimeric aptamer complexes and the utilization of photodissociable chain connectors, which enhanced the drug loading capacity and provided spatiotemporal control over release [163]. Recent studies by Zhu et al. have demonstrated that through simple biocompatible reactions, aptamer–drug conjugates containing multiple copies of drugs can be constructed, including various combinations of anti-cancer drugs, to augment anti-tumor effects [164].

Moreover, covalently linked aptamer–drug conjugates show promise in diverse cancer treatment regimens. He et al. developed a triterpenoid ketone ApDC targeting triple-negative breast cancer (TNBC), exhibiting high specificity and cytotoxicity against the MDA-MB-231 cell line [165]. Importantly, this triterpenoid ketone type ApDC exhibited significant anti-tumor efficacy in TNBC in vivo, with minimal side effects on healthy organs. Overall, covalent bonding offers a flexible and effective approach for utilizing nucleic acid aptamers in the field of drug delivery. Continued research on aptamer–drug conjugates will lead to the development of novel chemical strategies, further driving innovation in this field and expanding the possibilities for designing and optimizing efficient targeted drug delivery strategies.

#### 4.2.2. Noncovalent Conjugation

In addition to stable covalent bonds, noncovalent aptamer–drug coupling offers a simple yet effective drug delivery strategy for targeted therapy by leveraging programmable nucleic acid engineering. Previous reports have identified the 5′-(GC)-3′ or 5′-(CG)-3′ sites in dsDNA/dsRNA as exposed drug embedding sites [166]. In one approach, multiple Dox molecules are physically loaded into a long, repetitive drug-embedding dsDNA sequence, forming an aptamer–drug conjugate. By capitalizing on this feature, these conjugates act as ligands anchored to DNA nanosets [167]. The latest research by the Tan team explores a unique drug delivery platform using aptamer-modified DNA nanostructures. The sgc8 aptamer serves as a targeting molecule while the dsDNA nanostructure carries numerous Dox embedding sites, creating a specialized drug delivery “locomotive-boxcar” system. The synthesized sgc8c-NTrs exhibits excellent loading capacity, achieving a molar ratio of 50:1 for Dox:sgc8-NTr. This design holds promise for minimizing side effects and expanding the potential application areas of targeted drug delivery.

Zhu et al. introduced an innovative drug delivery system based on bispecific aptamers [167]. This system utilizes two aptamers, sgc8 and sgd5a, with drug insertion into dsDNA serving as the linker and drug carrier. Its uniqueness lies in its ability to identify two cancer subtypes with heterogeneous biomarkers, thereby overcoming many diagnostic and therapeutic complications. This study provided robust support for future clinical applications and underscored the potential of aptamers in the field of drug delivery.

**Figure 7:**
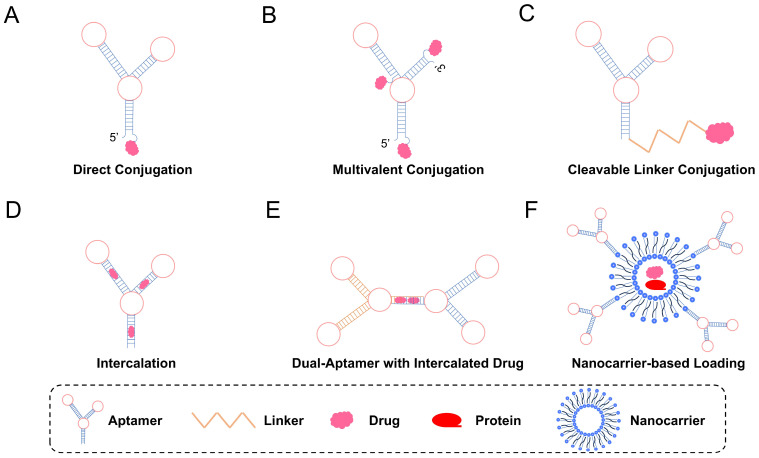
Examples of ApDC from covalent and noncovalent conjugation. (**A**) The drug is covalently attached directly to the aptamer without a linker, typically at the 5′ or 3′ end. (**B**) Multiple drug molecules are covalently attached to different sites of the same aptamer, enhancing therapeutic potency or enabling combination therapy. (**C**) A cleavable linker connects the drug to the aptamer, enabling triggered drug release under specific conditions (e.g., acidic pH, redox environment, or enzymatic cleavage). (**D**) The drug is noncovalently inserted between nucleobases of the aptamer via π–π stacking interactions, commonly observed with intercalating agents like doxorubicin. (**E**) Two aptamers are linked by a DNA duplex that noncovalently accommodates drug molecules. (**F**) The aptamer is noncovalently attached to the surface of a drug-loaded nanocarrier (e.g., liposome, polymeric nanoparticle, gold nanoparticle), serving as a targeting ligand for enhanced drug delivery.

Figure 7 systematically illustrates a variety of aptamer-based drug delivery strategies, categorizing them into covalent (Figure 7A–C) and noncovalent (Figure 7D–F) approaches.

### 4.3. Nanomaterial Drug Delivery

Due to their high drug loading capacity and enhanced permeability and retention effects in various tumors and inflammations, nanomaterials have emerged as promising drug carriers for therapeutic applications, achieving passive targeted drug delivery. By combining with active “targeting aptamers”, aptamer–nanocarrier conjugates offer numerous advantages. Particularly in the targeted delivery of chemotherapy drugs, researchers have explored various nanomaterials as carriers, including liposomes, micelles, polymers, DNA, gold, and magnetic nanoparticles [168]. Aptamer functionalization enhances the specificity of nanoparticles and has been reported in multiple studies, such as those on target cell imaging [169], active targeted therapy [170], or efficient cell sorting [171]. Among these nanocarriers, poly lactic-co-glycolic acid (PLGA) nanoparticles have been investigated for decades [172,173]. Due to their biocompatibility and biodegradability, PLGA has been FDA-approved for various clinical applications, including sustained drug release. To enhance cell, tissue, and disease specificity in targeted drug delivery, aptamer-functionalized PLGA nanoparticles have been extensively studied over the past decade. Additionally, polyethylene glycol has been integrated into these nanoparticles, self-assembled using amphiphilic PLGA-PEG diblock and PLGA-PEG-aptamer triblock polymers [174]. Functionalized PLGA-PEG nanoparticles possess sustained drug release, immune evasion, and specific targeting abilities. Notably, tumor-specific aptamers modified on nanocarriers improve drug delivery efficiency, thereby enhancing tumor treatment efficacy.

These nanoparticle–aptamer drug conjugates have effectively delivered cisplatin to PSMA-positive prostate cancer, with the cytotoxic drugs docetaxel and paclitaxel embedded in biodegradable polymers [175] or cisplatin precursors containing Pt(IV). These precursors can be converted into cytotoxic Pt(II) species through the intracellular reducing environment [176]. Cao et al. developed an innovative ligand–liposome delivery system using an anti-nucleolin aptamer sequence modified with a 12-thymine spacer at its 3′ end for modification [177]. They successfully encapsulated the hydrophilic dye calcein (for internalization monitoring) or chemotherapy drug cisplatin (for inducing anti-proliferative activity) in the core of liposomes. The researchers not only demonstrated the specific targeting effect of the aptamer system on breast cancer cells overexpressing nucleolin but also designed a complementary DNA to destroy the aptamer’s structure, thus achieving the reversal of drug delivery. Similarly, anti-nucleolin aptamers have been applied to functionalize liposomes containing doxorubicin as effective drug carriers [178]. This study provided important guidance for the further optimization of the aptamer–liposome delivery system [179].

The advancement of aptamer-driven drug delivery systems is anchored by four central pillars of innovation. Firstly, sophisticated modification strategies employing dynamically adaptive chemistries responsive to biological environments enhance the stability and responsiveness of aptamers. Secondly, the development of ‘smart’ delivery vehicles that integrate nanotechnology with environmental sensitivity enables precise, targeted drug release. Thirdly, the personalization of medicine through precision targeting leverages unique patient biomarkers for tailored therapy design and companion diagnostics. Lastly, the field of immuno-engineering focuses on mitigating immunogenicity and steering immune responses, potentially through aptamer-enabled immunotherapies and tolerance induction methods.

## 5. Conclusions and Future Perspectives

This paper has highlighted the significant advancements in nucleic acid aptamers, focusing on their theoretical foundations and innovative applications in therapy and drug delivery through SELEX technology.

Over the past three decades, nucleic acid aptamers have evolved into versatile molecular platforms, progressing from simple molecular recognition elements to functional agents in therapeutic intervention and precision drug delivery. Central to this evolution is the continuous advancement of SELEX technologies. Variants such as CE-SELEX, Cell-SELEX, Microfluidic SELEX, and the incorporation of NGS have markedly enhanced the specificity, efficiency, and throughput of aptamer selection. Particularly, the integration of NGS has transformed SELEX into a data-informed, iterative screening system, accelerating the identification of high-affinity, functionally relevant aptamers.

Aptamers exhibit a range of favorable characteristics, including high target specificity, low immunogenicity, ease of synthesis, and flexible chemical modifiability. These features render them attractive candidates for both therapeutic application and targeted delivery strategies. Their dual role—as therapeutic agents and as delivery vehicles—has been demonstrated in diverse disease contexts such as thrombosis, cancer, and viral infections. The development of aptamer–drug conjugates has further expanded their biomedical potential, enabling site-specific drug delivery, controlled release kinetics, and synergistic effects when combined with other modalities like siRNA or chemotherapeutics.

Despite these notable strengths, several limitations persist that impede clinical translation. Aptamers often suffer from limited in vivo stability, rapid renal clearance, and vulnerability to nuclease degradation. To address these challenges, various chemical and structural modifications—such as 2′-O-methylation, PEGylation, locked nucleic acids, and circularization—have been explored, alongside the integration of aptamers into nanoscale delivery systems to enhance stability and targeting precision.

From our perspective, aptamers represent a dynamic and evolving class of biomolecules with considerable promise in next-generation molecular medicine. While their inherent advantages establish a strong conceptual foundation, the successful clinical implementation of aptamer-based therapeutics will depend on the continued refinement of selection methodologies and robust preclinical validation. With ongoing innovation, aptamers are poised to play a central role in shaping the future of targeted therapy and precision diagnostics.

## Figures and Tables

**Table 1:** The comparative analysis of aptamers and protein antibodies.

Criteria	Aptamers	Antibody
Composition	Short single-stranded DNA or RNA oligonucleotides	Protein molecules composed of two heavy and two light chains
Molecular weight	~5–25 kDa	~150–180 kDa
Target range	Broad target recognition spectrum (e.g., peptides, proteins, small molecules, organic compounds, metal ions, viruses, bacteria, yeast, and mammalian cells)	Narrow target recognition spectrum (e.g., proteins, peptides, and carbohydrates)
Generation method	In vitro screening techniques	In vivo immunization or hybridoma technology
Affinity	High binding affinity	High binding affinity
Specificity	High specificity via sequence and structural optimization	High specificity for antigen epitopes
Chemical modification	Easily amenable to chemical modifications	Chemically less modifiable
Chemical conjugation	High chemical conjugation flexibility	Limited chemical conjugation capability
Degradability	Susceptible to nuclease degradation; modifiable for enhanced stability	Resistant to enzymatic degradation
Immunogenicity	Low or none immunogenicity	High immunogenicity

**Table 2:** Summary of representative aptamers: types, targets, stages, applications, and modifications.

Aptamers	Type	Target	Stage	Application	Modifications	References
Pegaptanib	RNA	VEGF165	Approved	AMD	2′-Fluoro, PEGylated	[61]
Pegpleranib	DNA	VEGF	Phase III	Combination therapy for AMD	PEGylated	[105]
Avacincaptad pegol	RNA	Complement C5	Approved	Geographic atrophy (dry AMD)	2′-Fluoro, 2′-O-methyl, PEGylated	[106]
HD1	DNA	Thrombin	Phase I	Anticoagulant (HIT therapy)	PDA	[72,73]
NU172	DNA	Thrombin	Phase II	Anticoagulant	None	[74]
ARC1779	RNA	vWF	Phase II	Thrombotic thrombocytopenic purpura	2′-Fluoro, 2′-O-methyl, PEGylated	[75,76,77]
BT200	DNA	vWF	Phase II	Thrombotic disorders (prevents arterial thrombosis)	PEGylated	[78]
DTRI-031	RNA	vWF	Phase I	Thrombotic disorders	2′-Fluoro, PEGylated	[79]
REG1 system	RNA	FIXa	Phase III	Reversible anticoagulation	dOxa	[83]
NOX-A12	RNA	CXCL12	Phase II	Cancer immunotherapy	PEGylated	[107]
TLS11a	DNA	Human hepatocellular carcinoma cells	Preclinical	Targeted therapy for hepatocellular carcinoma	FC protein	[100]
TLS1c	RNA	TLS/FUS protein	Preclinical	Amyotrophic lateral sclerosis and certain cancers	T15	[101]

**Table 3:** The solution strategies of aptamer-based therapeutics.

Aspect	Challenge	Solution Strategies
Aptamer stability	Susceptibility to nuclease-mediated degradation	1. Chemical modifications 2. Incorporation of unnatural nucleotide analogs 3. Encapsulation in nanocarriers or liposomes
Renal filtration	Rapid renal clearance due to low molecular weight	1. PEGylation or protein conjugation 2. Multivalent aptamer complex formation 3. Tissue-targeting ligand integration
Toxicity	Non-specific immune activation or off-target effects	1. Rational sequence design to minimize off-target binding 2. Comprehensive in vitro/in vivo toxicity assessment 3. Targeted delivery system development

## Data Availability

Not applicable.
